# Dermatofibrosarcoma protuberans: A case report of a rare, bulky tumor that was managed with surgical therapy

**DOI:** 10.4103/0973-6042.48432

**Published:** 2009

**Authors:** Ahmet Yılmaz, Erol Çenesizoglu, Ertuğrul Eğilmez, Safa Önel, Mehdi Muştu, Ahmet Cennet

**Affiliations:** Adana Numune Egitim ve Arastırma Hastanesi, Department of Orthopedics, Antakya, Turkey; 1 Dermatology Antakya, Turkey; 2 Mustafa Kemal University, Faculty of Medicine, Department of Radiology, Antakya, Turkey; 3 General Surgery, Antakya, Turkey; 4 Pathology, Adana, Antakya, Turkey

**Keywords:** Dermatofibrosarcoma protuberans, soft tissue tumor, surgical therapy

## Abstract

We report a case of this rare tumor and describe the surgical approach that we adopted and the clinical outcome; we also report his condition at 24 months' follow-up. Although treatment was with radical surgery, sufficient shoulder function could be obtained in our patient. We also describe the interesting MR findings of this tumor which correlated well with the histopathologic findings.

## INTRODUCTION

Dermatofibrosarcoma protuberans (DFSP) is a relatively rare, slow-growing, malignant mesenchymal tumor of the dermis. The tumor accounts for less than 0.01% of all malignancies.[1] The yearly incidence rate between 1982 and 2002, as per the population-based cancer registry in France, was reported as three cases per million population.[2] The neoplasm is locally invasive and relapses, due to insufficient excisions, are not rare. Remote metastases are also rare, but when it does occur the common sites are the lungs and the regional lymph nodes.[3,4]

DFSP usually begins as a polypoid protuberance or an indurated plaque on the skin. With time the tumor grows in size and ulcerations may appear on nodular projections on its surface. The tumor is mostly located on the trunk, with the other common locations being the head and neck. The size of the tumor is generally under 5 cm.[3,5,6] In the present case, the bulky (13 × 18 cm) tumor was located on the proximal arm, which is unusual. MRI of the lesion revealed multiple tail- like extensions into the deep muscle planes, which was also demonstrated by histopathology. This finding is useful during surgical planning.

We report a case of this rare tumor and describe the surgical approach that we adopted and the clinical outcome; we also report his condition at 24 months' follow-up. Although treatment was with radical surgery, sufficient shoulder function could be obtained in our patient. We also describe the interesting MR findings of this tumor which correlated well with the histopathologic findings.

## CASE REPORT

The patient (F.O.; 46-year-old male) was admitted to the Department of Orthopedics and Traumatology Service of Adana Numune Education and Research Hospital as 8562 protocol number on April 2006. He complained of a sizable, bulky tumor on the proximal part of his right arm, partially abutting on the shoulder; there was an ulceration present on the surface of the mass. According to the patient, he had first noticed a 1-cm nodule on his arm 5 years ago, which had grown gradually since then to attain its present size. He also explained that his dread of injections had prevented him from seeking medical aid earlier.

**Figure 1:**
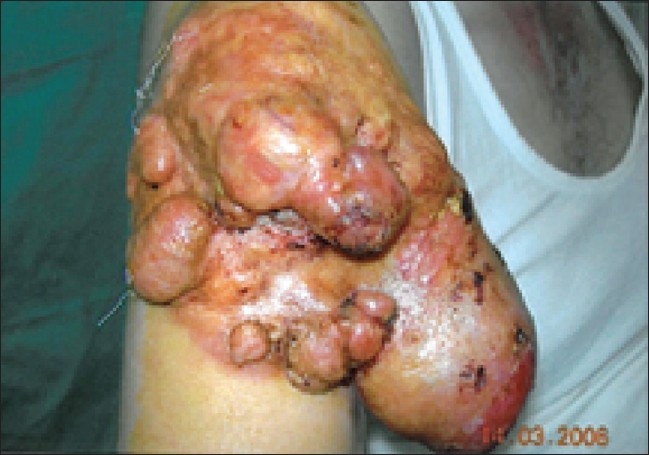
The morphologic appearance of the tumor

The physical examination revealed a huge mass on the proximal, anterolateral aspect of the right arm and shoulder; there were potato-like projections arising from the mass. The tumor was 13 × 18 cm in size; the overlying skin was of normal color, with hemorrhagic ulcerations on it [Figure 1]. The tumor was fixed to the deep tissues. Regional lymph nodes were not enlarged. Clinical examination did not reveal any other abnormality.

**Figure 2:**
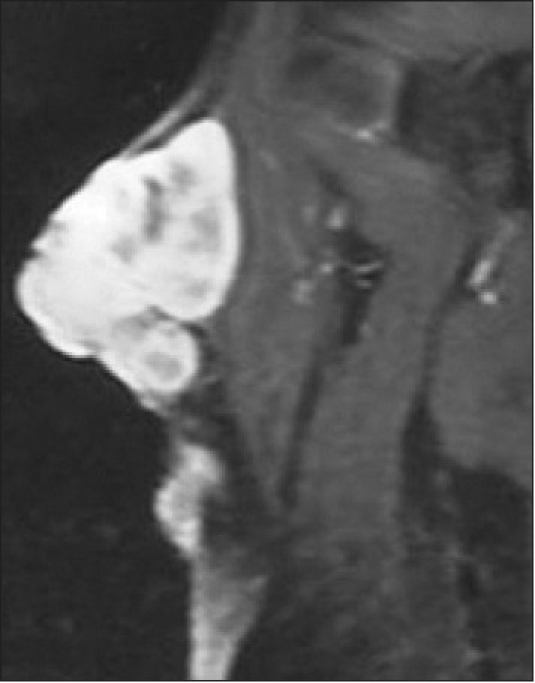
The T_2_-weighted, fat-saturated coronal MR image showed the solid mass with multilobulated, relatively homogenous high-signal-intensity pattern, with focal invasions in the deep muscle planes and cutaneus and subcutaneus tissues

**Figure 3:**
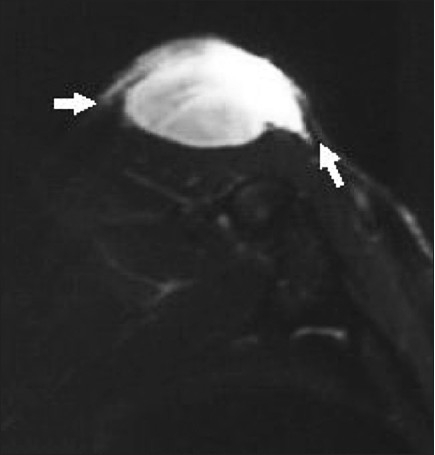
Tail-like projections in fibrosarcoma protuberans in fat- saturated T_2_-weighted axial MR image. The tumor with its high-signal-intensity pattern shows tail-like deep extensions (arrows) with some indefinite contours

**Figure 4:**
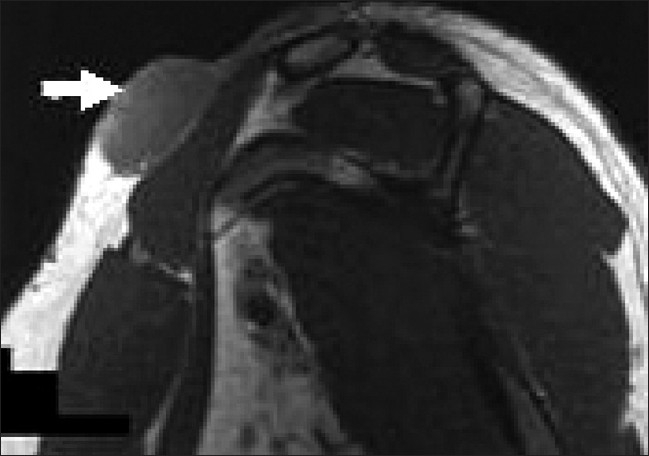
The mass lesion revealed a low-signal-intensity appearance with a tiny tail-like extension superiorly on T_1_-weighted images

The scout x-rays of the shoulder and proximal arm revealed a huge bulky soft tissue mass without involvement of the bony structures. The lung fields were normal on chest x-ray. MRI was done to evaluate the communications of tumor with neighboring structures and showed a multilobulated, relatively homogenous, high-signal-intensity solid mass with focal invasions at the deltoid muscle and deep extensions into the biceps, brachialis, and triceps muscles inferiorly; there was also infiltration of the cutaneus and subcutaneous tissues on fat-suppression images [Figure 2]. The mass was 13 cm in longitudinal diameter and extended 6 cm inferiorly. The dermal extension of the tumor had multiple tail-like projections up to 15 mm long. The imaging findings were strongly suggestive of malignancy and so we decided to operate. To the best of our knowledge, the focal invasions in the deltoid muscle and tail-like projections into dermis are important and new findings in the MR imaging of this tumor [Figure 3]. The mass lesion revealed a low-signal-intensity appearance with tiny tail-like extension superiorly on T1-weighted image [Figure 4]. Nuclear study revealed no abnormality. Laboratory studies were also within normal limits.

**Figure 5:**
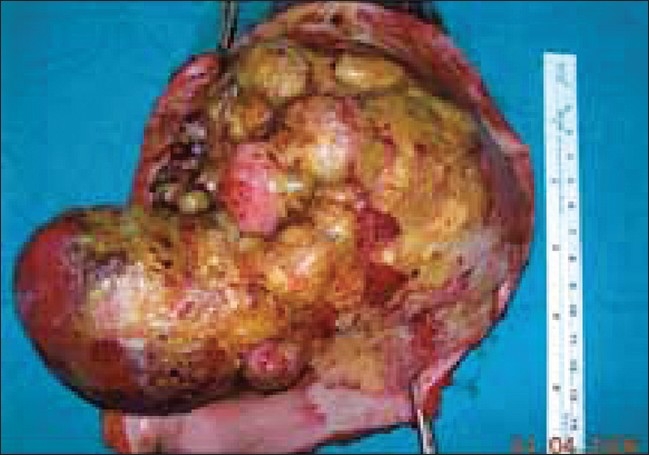
Per-operative tumor mass after resection

In view of the possibility of peripheral extension of the tumor tissue, a wide resection was planned so as to include more than a 3-cm margin of normal cutaneus tissue, at least 2 cm of the underlying normal tissue of the deltoid muscle, and also approximately 0.5 cm of noninfiltrated superficial layers of the other arm muscles [Figure 5]. We also decided to do a flap revision to cover the surgical defect 2 weeks later in order to evaluate the intact peripheral tissue.

**Figure 6:**
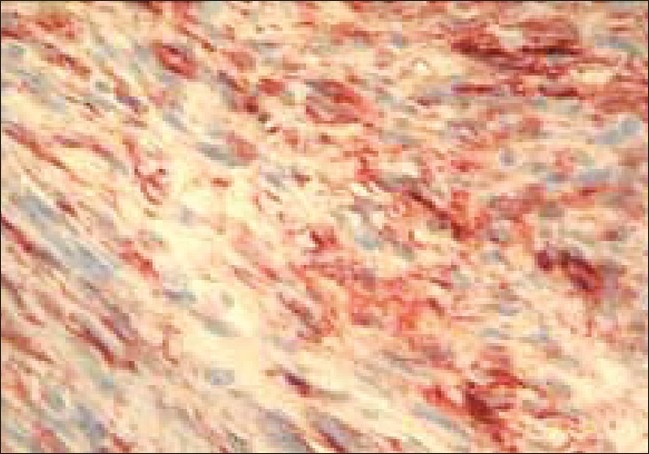
Immunohistochemical appearance (×40 100 HE)

The surgical procedure was carried out as planned and the surgical defect was covered. Macroscopic examination of the excised tumor revealed potato-like bulbous extensions on its surface. The cut surfaces were pink-white in color. Microscopic examination, which included histopathologic and immunohistochemical analysis with CD-34, confirmed the diagnosis of DFSP [Figure 6]. The surgical boundaries were found to be negative after operation.

**Figure 7:**
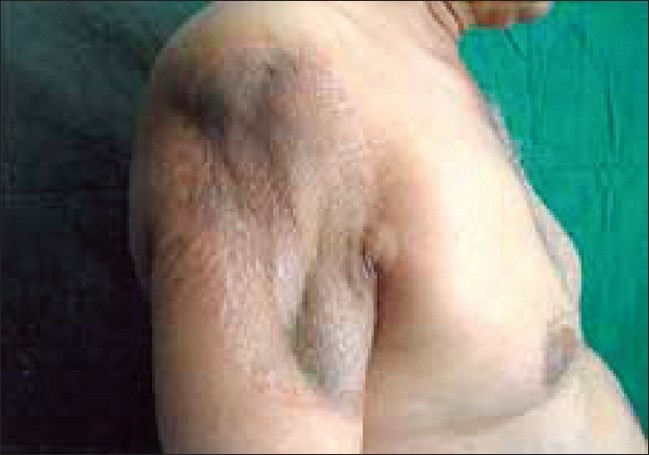
The clinical appearance of the shoulder of the patient 21 months after the operation

**Figure 8:**
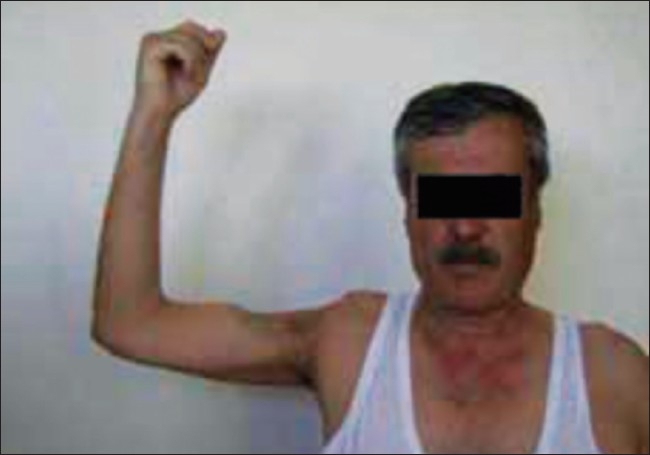
The clinical appearance of patient 24 months after surgery. Open space shoulder activity is shown

Three weeks after repair of the surgical defect, there was limitation of the range of movement of the shoulder, with only 20° abduction and minimal medial-lateral rotation. A physical therapy and rehabilitation program was prescribed, and after 3 months of an intensive exercise program, the performance of the shoulder had improved, with 90° abduction, 70° external rotation, and 25° internal rotation [Figures 7 and 8].

We followed up the patient every 3 months. The patient's general condition was very good at the last follow-up visit, 24 months after the surgery. No evidence of local relapse was seen, though there was a residual deformity at the operation site. No enlargement of cervical and axillary lymph nodes was present. Hematologic tests and radiographs of the shoulder, arm, and chest were normal. Follow-up MR examination was normal. Bone scintigraphy also showed no abnormality. At the last follow-up, the patient was actively using his arm and reported that he was carrying out his routine daily activities easily.

## DISCUSSION

DFSP was first described clinically in 1890. Darrier and Ferran described the tumor as 'progressive and recurrent dermatofibrosarcoma or skin fibrosarcoma.' The term 'dermatofibrosarcoma protuberans' was first used in 1925 by Hoffman.[3,7] The entity originates from the dermis and is a rare malignant dermal tumor. Although it can be seen at any age, it is found mostly between 30–50 years of age. Approximately 10% of patients are children. The incidence is 0.8–4.2 per million population. The tumor afflicts both sexes approximately equally.

The cause of DFSP is not known. However, cytogenetic investigations have shown tumor cells with chromosomal anomalies together with mononuclear cellular origin. Some studies also describe the occurrence of DSFP at vaccination sites, in the scars of surgical incisions or of dermal burns, and in areas affected by leishmaniasis. Histologically DSFP is composed of monomorphic, benign appearing spindle cells. These cells are organized in a storiform or rush mat pattern and align perpendicular around the vessels in central locations.[8‐11] A rare type of DFSP is characterized by dendritic cells that contain melanin and is known as 'pigmented DFSP' or 'Bednar tumor.' The clinical findings, localization, and microscopic properties of this variant are similar to that of the common type of DFSP.

Roughly 50% of DFSP lesions are found on the trunk. The rest are found on the proximal extremities and the head and neck region. Involvement of hands and feet is rare.[3,5‐9,11] The clinical findings are related to the duration for which the tumor has been present. In the early phase, the tumor is an asymptomatic, small, indurated plaque. It may be pink, purplish-red, or similar to the surrounding normal skin in color. Later, rigid polypoid nodules appear, and as the nodules grow ulcerations develop on the surface. These lesions are typically fixed to the dermis. However, they are freely mobile on the deep tissues. Fixation to the deep structures has been seen at late stages or in case of local relapse.[3,9] The early lesion usually appears as a solid nodule, as a soft depressed plaque, or as a rounded yellowish sclerotic disc.

Multiple finger-like projections grow within the nodules at the periphery of the tumor mass. The DFSP with its finger-like projections is frequently disseminated to beyond to the level of vision. Insufficient excisions of these projections were thought to be responsible for the high relapse rate.[5] The tumor invades laterally through the fascicles of the dermis over a period of many years, which may vary from half a year to 30 years. After this period, the tumor enters the growth phase and infiltrates the subcutaneus tissues and deep fascial structures.[3,7] Satellite nodules coalesce to form a bulky mass, producing the typical protuberant appearance of well-developed lesions. Pain and increased sensitivity might indicate the early stages of the period of rapid growth. As the mass grows aggressively, the skin becomes stretched and thin, ulcerations may form, and there may be bleeding. In some untreated cases, dermal infarcts can be seen on the surface of bulky tumors. Reported diagnostic survey was up to 10 years. The average tumor size has been reported to be under 5 cm and, according to the literature, tumors of 10 cm or more are extremely rare.

The metastases rate is reported to be under 5% in DFSP. Metastases may occur many years after the onset of the disease and are mostly to lungs, followed by the regional lymph nodes; the visceral organs and bones are rarely affected. Generally, distant metastases is seen after recurrent local relapses.[3,5‐9] However, metastases to the lungs and pancreas have been reported in the absence of local recurrence.[5,12] The most likely explanation for the occurrence of local relapse is insufficient local resection with positive boundary tissue on histopathology. Poor prognostic factors are older age, increased activity, and advanced period.

The tumor size and mobility is evaluated by physical examination. Blood tests are not useful for diagnosis. The scout x-rays reveal only a soft tissue mass without any other additional findings. MRI is useful to demonstrate deep tissue invasion, especially in the advanced stages. Nuclear study is useful for ruling out bone invasion. Though x-rays must be taken in all cases, computed tomography is not indicated if there is no bone invasion.

Histologically, DFSPs should be differentiated from fibrohistiocytic neoplasms and lesions such as malignant fibrous histiocytoma, atypical fibrosarcoma, dermatofibrosarcoma, infantile myofibromatosis, nodular fasciitis, keloid, and hypertrophic cicatrix, all of which may have similar pathologic findings. In the immunohistochemical investigations, the CD-34 marker is positive in all the cut slices. Other fibrohistiocytic lesions usually show negative results in CD-34 expression.[4,5,7,8,13,14]

The preferred therapy of DFSP is radical (wide) surgical excision. The technique should include resection of a 3-cm margin of skin beyond the borders of the tumor and should include the fascia and even muscle tissue if necessary to attain negative intact borders histopathologically.[2‐6,8,15,16] Local relapse rate is below 10% after extensive excision. When the margin of surrounding skin was less than 3 cm margin the local relapse rate was around 50%.[3,10] Relapses were mostly seen within the first 2 years. Extensive surgical excision of DFSP tumor is not possible on the hand, foot, or face areas and, in these locations, Mohs micrographic controlled surgical procedure is done.[3‐5,7,8,13]

Radiotherapy is of limited value in the treatment of DFSP. However, it might have some role to play when the resection border is positive or when extensive excision is not possible due to cosmetic or functional difficulties.[3,5,7,8] Imatinib mesylate, a drug used in chronic myelogenous leukemia, has been successful in metastatic disease and/or relapses of the entity.[17]

It has been reported that some cases of DFSP relapse as late as 5 years after surgery. A major weakness of our study is that the follow-up time is only 2 years. However, literature reports do suggest that recurrences mostly occur within the first 1 or 2 years.

We performed a wide resection in our case, excising a 3-cm margin of intact tissue, which also included normal underlying muscle. Radiotherapy and chemotherapy were not used because the incision borders were negative on histopathology. Today our patient lives a normal life, without evidence of relapse or metastases. The history and clinical situation of our patient corresponds with that of patients in previous reports. In the literature, the average size of these tumors is reported as being under 5 cm. In our patient the tumor was 18 × 13 cm in size, which is exceptional; despite the large size there were no metastases.

To conclude, wide radical excision is the preferred surgical method for therapy of DFSP.
